# Supplementary material for: Stakeholder priorities and conceptualization of One Health: Insights from fuzzy cognitive mapping and grounded theory
Source: PLoS One. 2026 Mar 2;21(3):e0338167. doi: 10.1371/journal.pone.0338167 (PMC12952644; doi:10.1371/journal.pone.0338167)
Supplement: Attachment — Submitted filename: Response_to_Reviewers.docx [file pone.0338167.s003.docx]

**Response to Reviewers**

Manuscript ID: ONE-D-25-61857
Title: Stakeholder priorities and conceptualization of One Health: Insights from fuzzy cognitive mapping and grounded theory

Dear Dr. Ogola,

We sincerely thank you and the reviewer for your careful evaluation of our manuscript and for the constructive feedback. We have carefully addressed all comments and believe the manuscript has been strengthened as a result.

Below, we provide a point-by-point response to your comments and Reviewer #1. As noted below, Reviewer #2’s comments appear to refer to a different manuscript.

Academic Editor and Journal Requirements

1. Formatting Requirements
We have revised the manuscript to ensure full compliance with PLOS ONE style and formatting guidelines.

2. Clarification of Verbal Consent Documentation
We have updated the manuscript with the following changes (Page 14, lines 246-251): “Prior to each mapping session, participants were informed about the purpose, procedures, data handling, confidentiality, and the voluntary nature of participation. Verbal informed consent was obtained from all participants. Consent was documented by the research team at the time of data collection and witnessed by EFG or CK, in accordance with the approved Tufts IRB study protocol.”

3. Funding Information in Manuscript
All funding information has been removed from the manuscript body and retained only in the Funding Statement section of the online submission form.

4. Grant Information Consistency
We reviewed and confirmed that grant information is now consistent across all sections of the submission.

5. Role of the Funder Statement
We included the following statement in the cover letter: “This research was funded by a Cummings Foundation grant (V710458) awarded to H.J.A. The funders had no role in study design, data collection and analysis, decision to publish, or preparation of the manuscript.”

6. Copyrighted Map Image (Figure 1)
We removed the original copyrighted basemap and replaced Figure 1 with a version that complies with CC BY 4.0 licensing requirements.

7. Removal of Figures from Manuscript File
All figures have been removed from the manuscript file and uploaded separately as individual TIFF files. Figure legends remain in the manuscript.

8–9. Table Numbering Issues
Table numbering has been corrected throughout the manuscript. **Table 5 starting on page 16 is now references in the text. There is no Table 6.**

10–11. Reference List Review
The reviewer comments did not include recommendations to cite specific works.

**Reviewer #1**

Comment 1: Clarify novelty of proposed theory (OHGT)
We expanded the Discussion to more explicitly articulate the novelty and broader applicability of One Health Grounded Theory (OHGT), clarifying its theoretical contribution beyond the immediate case study (Page 28, lines 543-559).

Comment 2–3: Sample size variation and limitations
We revised the Study Limitations section to explicitly address how sample size variation may affect concept saturation, centrality analysis, and structural stability across stakeholder maps (Pages 33-34, lines 660-688).

Comment 4: Reduce repetition
Thank you for this comment. Without more specific details regarding what repetition the reviewer is referring to it was hard to know what to change. However, we have made the following revisions with the intention of reducing repetition:

1) We deleted the last two paragraphs of the introduction which included information that was repeated in the Methods section (e.g., sampling and participant recruitment).
2) We deleted the “Study Site Selection,” as this presents information that is included in other parts of the Methods and Discussion section.
3) Removed any reference to the ethics process (Page 8) as this is included in the “Ethics Statement” (Page 12).
4) Finally, we edited the discussion to remove any references to results.

Comment 5: Clarify FCM figures
We added a concise overview of FCM at the beginning of the Methods section and ensured that figure legends clearly explain interpretation of node size, edge color, and arrow thickness. Adjacency matrices are included as supplementary material.

Reviewer #2

The comments provided under Reviewer #2 refer to a study involving the Smartphone Addiction Scale (SAS-SV) and logistic regression analysis, which is unrelated to our manuscript. We respectfully believe this review was inadvertently assigned from another submission.

Data Availability

Adjacency matrices for each stakeholder group are provided in the supplementary material. Qualitative transcripts are not publicly available due to participant confidentiality and IRB restrictions; however, coding structures and analytical procedures are described in detail in the Methods.

Conclusion

We greatly appreciate the constructive feedback provided by the Academic Editor and Reviewer #1. The revisions have strengthened the manuscript’s clarity, transparency, and theoretical contribution. We believe the revised version more clearly articulates its empirical and methodological contributions to participatory One Health research.

Sincerely,
Evan Griffith, DVM, MPH, MS
On behalf of all co-authors
